# Advances in nanomaterial-targeted treatment of acute lung injury after burns

**DOI:** 10.1186/s12951-024-02615-0

**Published:** 2024-06-18

**Authors:** Shuo Zhang, Xinyu Zhao, Yuhao Xue, Xianwen Wang, Xu-Lin Chen

**Affiliations:** 1 Department of Burns, The First Affiliated Hospital of Anhui Medical University, Hefei, 230022 P. R. China; 2 School of Biomedical Engineering, Anhui Medical University, Hefei, 230022 P. R. China

**Keywords:** ALI, Nanomaterials, Burn, Targeted treatment, Camouflaged nanoparticles

## Abstract

Acute lung injury **(**ALI**)** is a common complication in patients with severe burns and has a complex pathogenesis and high morbidity and mortality rates. A variety of drugs have been identified in the clinic for the treatment of ALI, but they have toxic side effects caused by easy degradation in the body and distribution throughout the body. In recent years, as the understanding of the mechanism underlying ALI has improved, scholars have developed a variety of new nanomaterials that can be safely and effectively targeted for the treatment of ALI. Most of these methods involve nanomaterials such as lipids, organic polymers, peptides, extracellular vesicles or cell membranes, inorganic nanoparticles and other nanomaterials, which are targeted to reach lung tissues to perform their functions through active targeting or passive targeting, a process that involves a variety of cells or organelles. In this review, first, the mechanisms and pathophysiological features of ALI occurrence after burn injury are reviewed, potential therapeutic targets for ALI are summarized, existing nanomaterials for the targeted treatment of ALI are classified, and possible problems and challenges of nanomaterials in the targeted treatment of ALI are discussed to provide a reference for the development of nanomaterials for the targeted treatment of ALI.

## Highlights

To summarize the mechanisms and pathophysiological features of ALI after burn.

To classify the targets for the treatment of ALI.

Describe the types of nanomedicines used to treat ALI.

These findings suggest the prospects and outlook for nanomaterial-targeted therapy for ALI.

## Introduction

Burns are tissue damage caused by heat (including hot liquids, steam, hot gases, flames, hot metals, etc.), which mainly affects the skin or mucous membranes and, in severe cases, subcutaneous or submucosal tissues such as muscles, bones, joints, and even internal organs [1, 2]. The lung is the organ with the highest and first highest incidence of insufficiency after severe burns [3]. Pathological changes in the lungs are found in 30–80% of burn patients [4, 5], and the main mechanism is destruction of vascular endothelial cells and alveolar epithelial cells, leading to damage of the alveolar capillary barrier, pulmonary edema, pulmonary hemorrhage, and severe gas exchange disorders. Burn patients with burns affecting more than 30% of the total body surface area (TBSA) often experience acute lung injury (ALI) [6]. Severe burns cause body damage mainly from traumatic stimuli, direct thermal aggression, and inadequate tissue perfusion, and these multiple stimuli can lead to secondary damage, such as an excessive inflammatory response, oxidative damage, stress, apoptosis, and immunomodulatory damage, which ultimately leads to ALI [7–9].

According to the literature, ALI has a mortality rate ranging from 35 to 40% and is very difficult to treat [10, 11]. Despite many years of exploration and development of a variety of clinical drugs, due to the difficulty of most drugs reaching the lungs, coupled with the ease of distribution throughout the body resulting in toxic side effects, limiting their clinical application, in addition to mechanical ventilation in the clinic, there are currently no other effective treatments available [12, 13].

Nanomaterials have important anti-inflammatory and antiapoptotic effects and promote immunomodulation due to their artificially modifiable and modifiable surface properties [14–16]. These methods have the following main advantages: (1) greater efficacy and less toxicity; (2) greater stability, solubility, and circulating half-life and can be used to artificially control drug loading; (3) molecular targeting can be accurately applied to the target; (4) they can be combined with imaging to obtain more sensitive and typical imaging results [17]; and (5) real-time monitoring of the distribution of drugs in the body and the location of dispersion can be performed for real-time feedback on the effect of drugs in the body [18]. With the deepening knowledge of the mechanism underlying ALI occurrence, scholars have developed several drug-carrying or nondrug-carrying nanomaterials based on lipids [19], peptides, organic polymers [20], extracellular vesicles [21, 22], and inorganic nanoparticles [23, 24] that can specifically reach the lungs for the treatment of ALI in response to the characteristics of pathophysiological features and immune modulation.

**Fig. 1:**
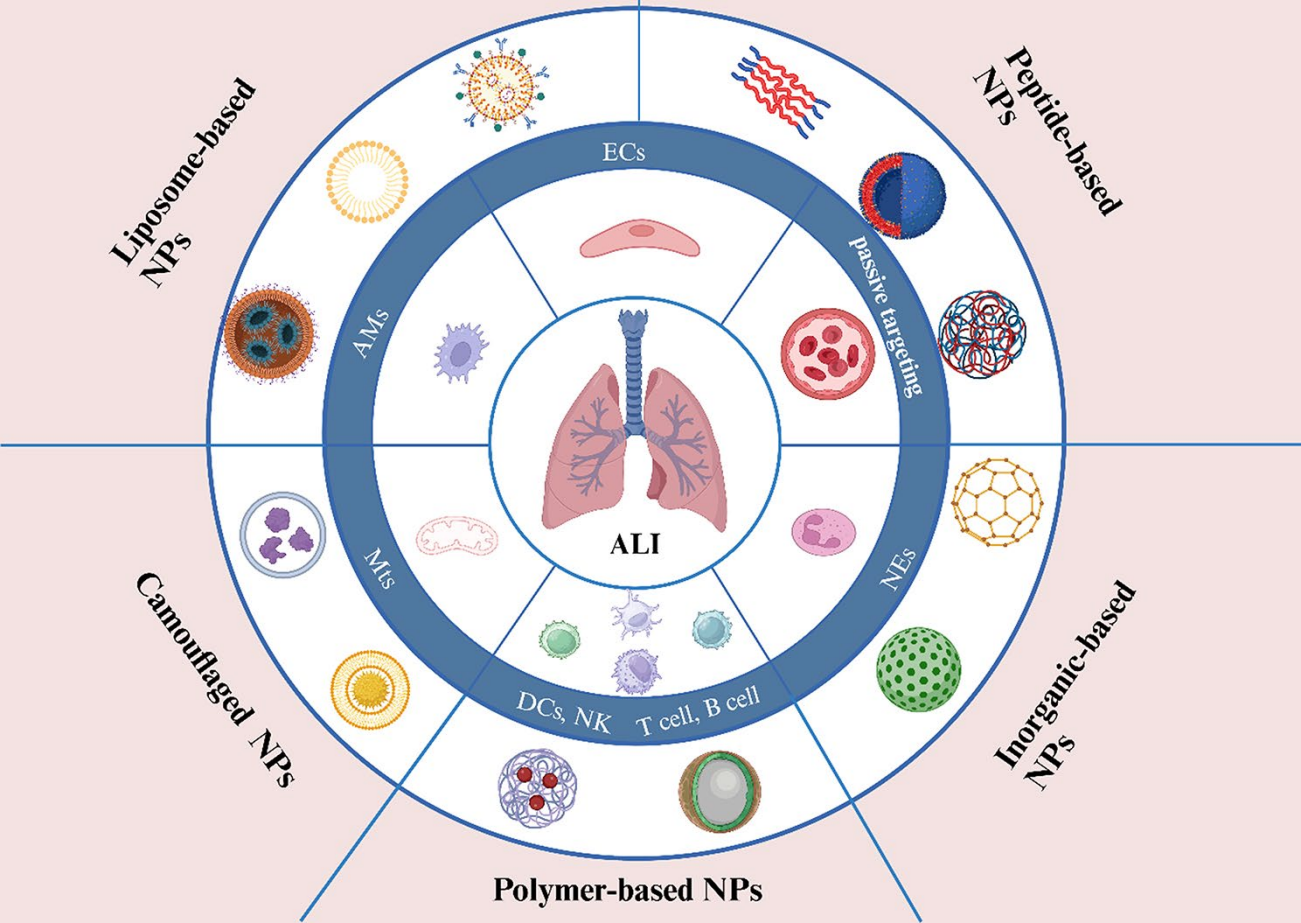
Schematic strategy for the nanomaterial-targeted treatment of ALI.

Although there are reviews describing the pathological features of ALI and the progress of multiple nanomaterial drug-targeted therapies for ALI [25–27], the mechanism of therapeutic targeting needs to be constantly updated as the understanding of the mechanisms of postburn ALI development deepens, especially the role played by immune modulation in ALI. There is no review that systematically summarizes the advantages, disadvantages and challenges encountered in targeting multiple nanomaterials for the treatment of ALI, such as how to eliminate immunity. In this review, first, the mechanisms and pathophysiological features of ALI occurrence after burn injury are reviewed, potential therapeutic targets for ALI are summarized (e.g., passive targeting, ECs, NEs, AMs, Mts, immune metabolism), existing nanomaterials for the targeted treatment of ALI have been identified (e.g., liposome-based NPs, polymer-based NPs, peptide-based nanoparticles, inorganic-based NPs, camouflaged NPs), and the possible problems and challenges associated with the use of nanomaterials for the targeted treatment of ALI are discussed (Fig. 1). In addition, future research directions for nanomaterial-targeted treatment of acute lung injury are also presented.

## ALI after burning

### Mechanisms of ALI after burn injury

**Fig. 2:**
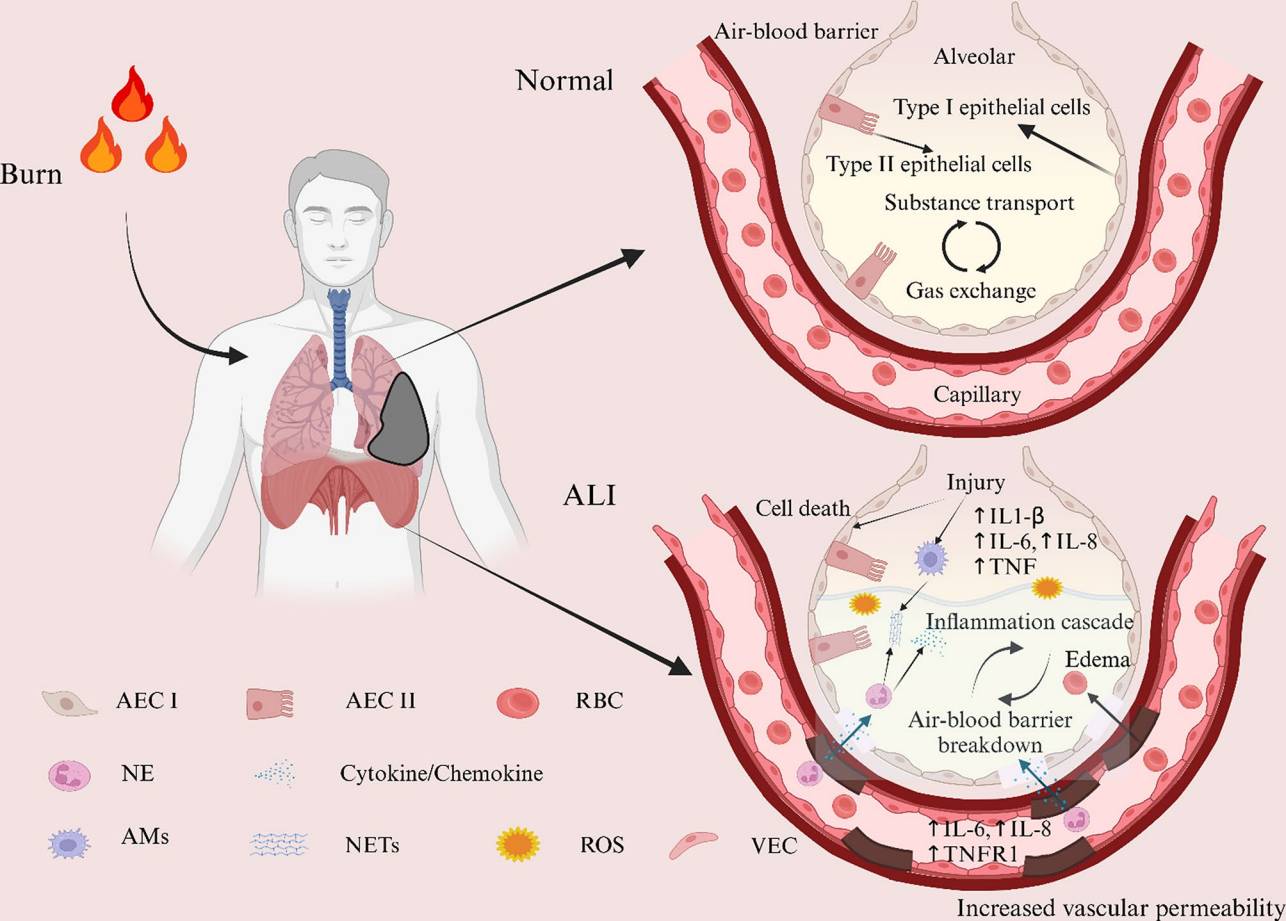
Mechanisms and changes in the internal environment of ALI after burn injury

ALI after burns is a common and serious complication of burns, and its mechanisms are complex and varied: (1) Burns lead to direct tissue destruction and necrosis, releasing large amounts of intracellular and extracellular biologically active molecules, such as cytokines, inflammatory mediators, and oxygen free radicals [28], these substances activate immune cells and other inflammatory mediators, causing an inflammatory response in the lungs; (2) After burns, the body undergoes a systemic inflammatory response, prompting the release of inflammatory mediators such as interleukin-1 (IL-1), tumor necrosis factor-alpha (TNF-α), and interleukin-6 (IL-6) in the bloodstream, and these inflammatory mediators can spread rapidly through the bloodstream to the lungs, activating inflammatory cells in the lungs and leading to inflammatory reactions in the lung tissue [29]; (3) Burns cause damage to the vascular endothelium and an increase in vascular permeability, leading to impaired pulmonary microcirculation, which can cause leakage of intravascular fluid and the formation of pulmonary edema, as well as making it easier for inflammatory mediators to penetrate into the lung tissue [30]; (4) Burns cause the generation of oxygen free radicals, a highly reactive class of oxidizing molecules produced during tissue hypoxia and reperfusion, which exacerbate the inflammatory response and cause direct damage to cellular and tissue structures [31]; (5) alveolar surface-active substances (ASASs) are lubricants that help prevent alveolar collapse, and in ALI, inflammation and edema may lead to disruption of ASASs, which may in turn affect respiratory function; (6) in ALI, epithelial and endothelial cells may undergo apoptosis, a controlled process of cell death, and apoptosis may damage lung structures and cause an inflammatory response; (7) The immune system may be abnormally activated in ALI, leading to an excessive inflammatory response and damage to lung tissue. ALI after burn injury is a complex pathophysiologic process that includes direct thermal traumatic effects and a systemic inflammatory response [32, 33]. Together, these factors lead to lung inflammation, edema, microthrombosis, and tissue damage, ultimately triggering ALI (Fig. 2).

### Stages of ALI development after burn injury

**Fig. 3:**
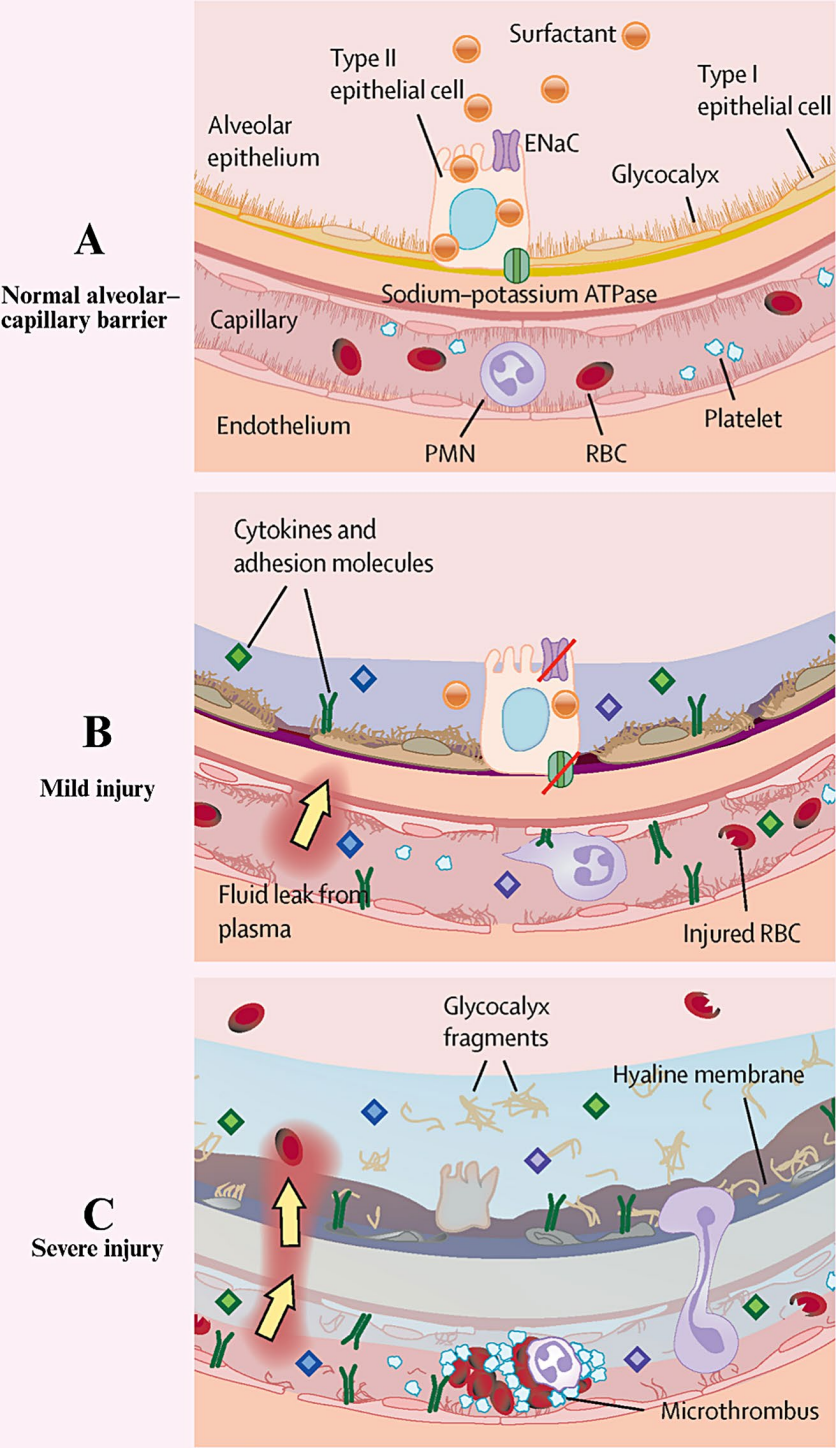
Progress in the occurrence of ALI. **(A)** The normal alveolar–capillary membrane is a barrier that prevents the development of alveolar edema. With the progression from mild **(B)** to severe injury **(C)**, the alveolar–capillary barrier becomes more permeable, leading to alveolar edema. adapted with permission from ref [40].,copyright 2022, Elsevier

ALI is pathophysiologically characterized by decreased lung volume, reduced lung compliance, and dysregulated ventilation/blood flow ratios. It clinically manifests as progressive hypoxemia and respiratory distress or even multiorgan failure [34–37]. The stages of ALI development include the following [38–40]: phase I, injury/activation. The initial injury is caused by a number of factors, such as direct lung trauma, infection, smoke inhalation, and toxin exposure. Chemical signals that cause inflammatory responses and cellular damage are released, leading to the accumulation of inflammatory cells and mediators. Phase II, Maintenance/Maintenance: Cellular inflammation: inflammatory cells (e.g., macrophages and neutrophils) are activated, releasing inflammatory mediators and triggering additional inflammatory responses. Vascular endothelial cells and alveolar epithelial cells are damaged, and their permeability is increased, leading to inflammatory cells entering the alveoli. Phase III, destruction/repair (destruction/resolution): (1) Alveolar edema: Plasma leakage leads to alveolar edema, which affects gas exchange. (2) Inflammatory response: A continuous inflammatory response may lead to destruction of lung tissue (Fig. 3).

### Treatments for ALI after burn injury

ALI after burn injury is a serious complication among burn patients, and treatment requires a combination of the burn itself and the particular circumstances of the ALI [41]. The following are the general principles of a treatment program for ALL after a burn injury [42–44]: (1) early supportive treatment; (2) mechanical ventilation and lung protective strategies; (3) fluid management: limited fluid intake to prevent further exacerbation of pulmonary edema; (4) infection control: antibiotic therapy and early antibiotic treatment for potential infection, especially at burn wounds; (5) supportive circulatory system: use of vasoactive drugs to maintain blood pressure when necessary; and (6) nutritional support.

A variety of drugs, such as biomolecule drugs composed of chemicals [45–47], antibiotics [48, 49], natural products [50], peptides [51], and genetic material [52, 53], have been clinically proposed for the treatment of ALI. In clinical trials, various drugs have been shown to alleviate lung injury, but the mortality rate of patients with ALI remains high. It is inextricably linked to the poorer water solubility and greater toxicity of the drugs [54, 55]. Currently, there is no effective treatment other than mechanical ventilation, and exploring drugs with low toxicity and a high targeting rate has become urgent.

## Multiple targets for treating ALI

Nanocarriers can deliver drugs for the treatment of ALI to the site of lung injury, which not only improves efficacy and increases drug utilization but also avoids toxic side effects [56, 57]. There are two main types of targeted agents for the treatment of ALI: passively targeted and actively targeted agents [58, 59]. Passively targeted agents rely primarily on extravasation through leaky vasculature and subsequent inflammatory cell-mediated sequestration (ELVIS) for targeted delivery of drugs to the site of ALI. The pulmonary ELVIS effect is an increase in the permeability of the local vascular system due to the disruption of the interstitial, vascular, and alveolar structures of the lungs, which in turn facilitates the passage of drugs or nanoparticles through the vascular wall to reach the diseased part of the lung tissue [60]. Actively targeted drug delivery systems can be selected to modify drugs or carriers with specific ligands to enhance the therapeutic efficacy of the drugs. Actively targeted therapeutic strategies for ALI focus on endothelial cells, inflammatory neutrophils, macrophages, immunomodulatory cells, and damaged mitochondria [61, 62]. Currently, based on the pathophysiological characteristics of ALI, researchers have developed a variety of targeted nanomedicine formulations with different carrier nanomaterials and functional group modifications, which are summarized as follows.

### Passive targeting

**Fig. 4:**
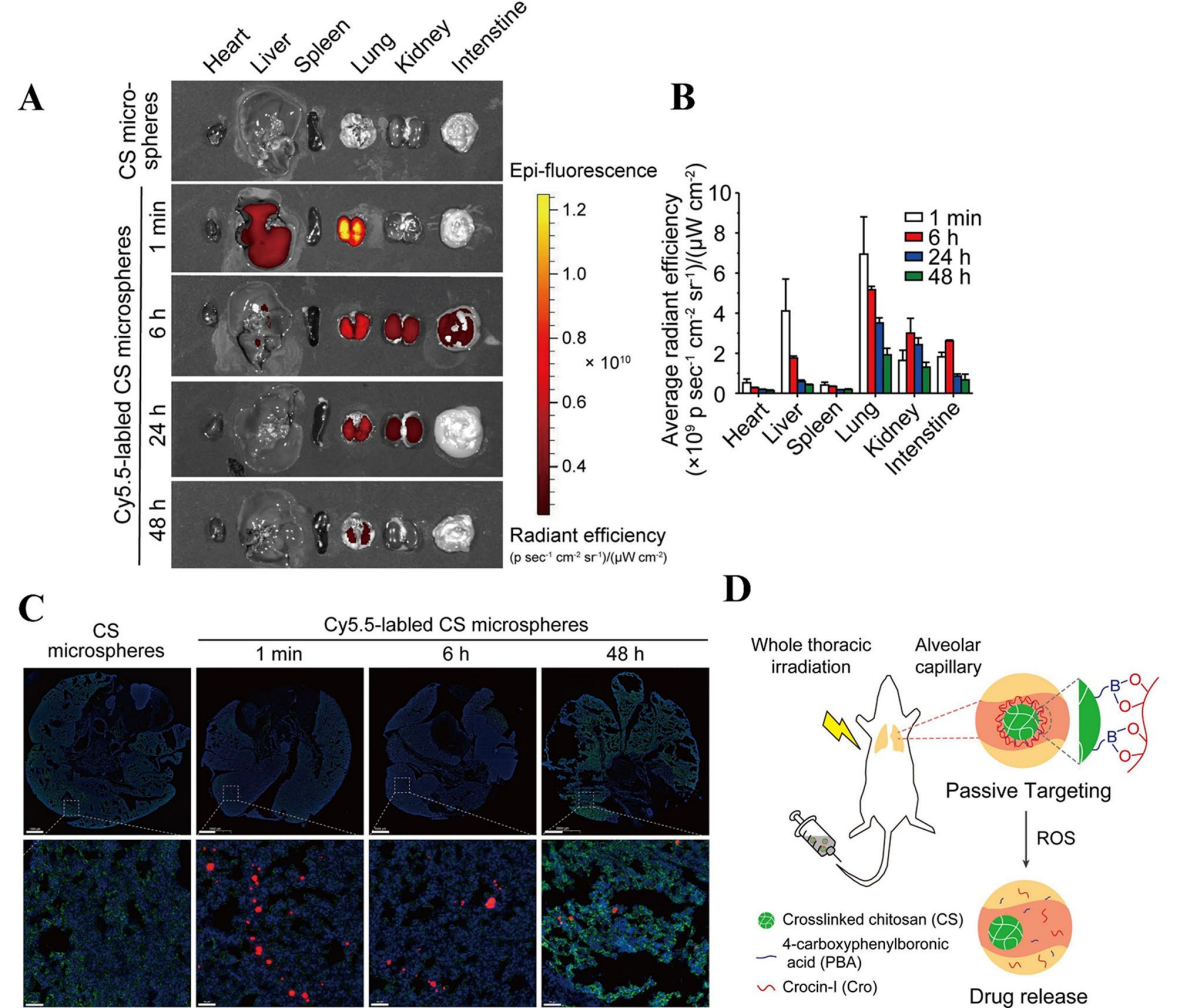
Passive lung targeting of CS-PBA-Cro microspheres after intravenous injection. **(A)** Representative fluorescence images and **(B)** quantitative analysis of label-free or Cy5.5-labeled CS microspheres in the main tissues at different time intervals after intravenous injection (1 min, 6 h, 24 h and 48 h). **(C)** Distribution of label-free or Cy5.5-labeled CS microspheres in cryosections of lungs after intravenous injection. **(D)** Graphical abstract. adapted with permission from ref [64]., Copyright 2023, Elsevier

After severe burns, lung tissues are damaged and necrotic, releasing a large number of intracellular and extracellular bioactive molecules, leading to vascular endothelial cell damage and increased vascular permeability. At this time, the fluid in the alveoli will hinder drug absorption, but at the same time, the ELVIS effect can also be utilized to design corresponding drug carriers to target the lungs to perform their functions. In the normal lung environment, endothelial cells act as barriers between normal tissues and blood, allowing the extravasation of small molecules while restricting the passage of large molecules and cells. At sites of inflammation, however, endothelial cells become abnormal and allow the passage of macromolecules or cells [63]. This microenvironment provides conditions for greater access of drugs and drug carriers to lung tissue. If passive targeting is utilized to improve drug efficacy, researchers need to consider the development of new drug carrier materials that allow more of the drug to be retained in lung tissue. For example, W. Lu et al. [64] designed injectable chitosan microspheres synthesized from the natural antioxidants crocin-I and 4-carboxyphenylboronic acid. Administered via tail vein injection to mice, the drug could not only be targeted to mouse lungs by passive targeting but also act for a longer period of time, improving the efficacy of crocin-I in the treatment of lung injury. (Fig. 4). Although passive targeting strategies have been widely used and studied, their targeting ability is still insufficient; thus, more precise targeting is needed for ALI treatment.

### Active targeting agents

#### Endothelial cells (ECs)

Recent studies have identified pulmonary ECs as the main target cells for the development of ALI due to various pathogenic factors [28]. Pulmonary ECs are located on the medial side of blood vessels and not only play a role as a selective permeability barrier but also have important endocrine functions; these functions are important for the regulation of vasodilatation and permeability, antiplatelet aggregation, and maintenance of the integrity of the vascular wall [65, 66]. Pulmonary ECs are important target and effector cells that mediate ALI, and the barrier and endocrine functions of endothelial cells are altered in the early stage of ALI, as evidenced by an increase in vascular permeability and excessive release of various inflammatory mediators [67]. Therefore, inhibiting EC activation and improving endothelial barrier function by designing nanomedicines targeting ECs are crucial for the treatment of ALI.

#### E-selectin

In ALI, endothelial E-selectin plays an important role. E-selectin is one of the major members of the selectin family of adhesion molecules and is expressed mainly in ECs [68]. E-selectin primarily mediates the first phase of leukocyte adhesion to vessel walls, the reversible adhesion phase. It mediates weak adhesion of leukocytes in contact with vessel walls by binding to its corresponding ligand. Leukocytes adhere to vascular endothelial cells through adhesion molecules and interact with them to produce a large number of inflammatory mediators, which kill invading pathogens [69]. However, elevated E-selectin strongly attracts leukocytes to the bloodstream, and the release of various oxygen radicals as well as inflammatory factors by leukocytes aggregated at the site of injury or inflammation can also lead to the amplification and uncontrolled control of the local inflammatory response, exacerbating tissue damage and organ dysfunction [70].

**Fig. 5:**
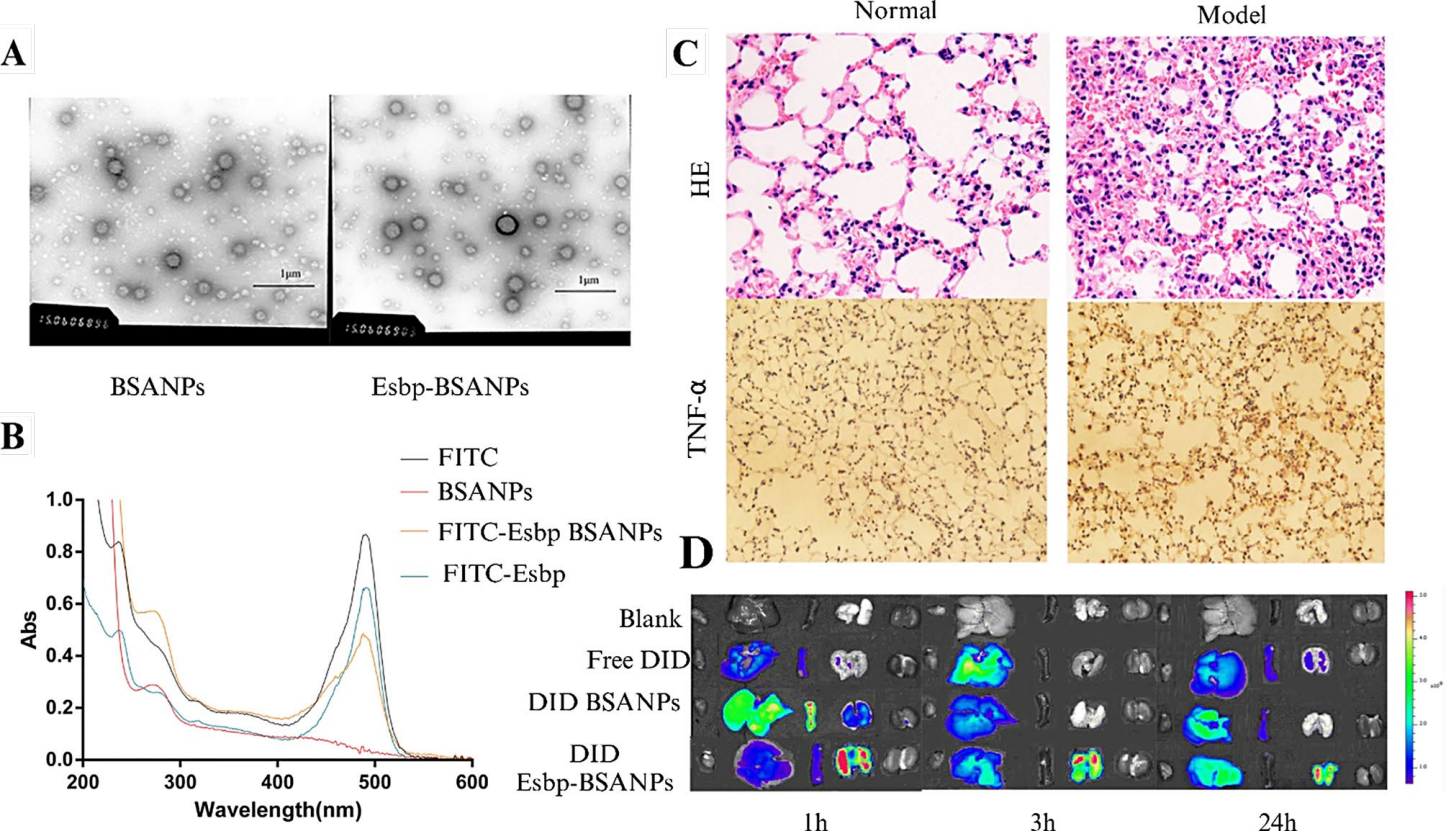
FITC-Esbp-BSANPs can target the lungs in an established ALI mouse model, and the nanoparticles are distributed in vivo. **(A)** TEM image; **(B)** UV images of the hydrolysate of Fitc, Fitc-labeled Esbp, DXM-loaded BSANP hydrolysate, and DXM-loaded Fitc-Esbp-BSANP hydrolysate from 200 to 600 nm. H&E staining of lung sections **(C)** of mice (in the normal group and inflammatory group) and in vivo organ images **(D)** from left to right: heart, liver, spleen, lung, and kidney) of mice in the inflammatory group. adapted with permission from ref [70].,copyright 2019, SPRINGER.

An E-selectin-binding peptide is a peptide with high affinity for E-selectin that binds specifically to E-selectin. Y. Liu et al. [70] reported the ability of bovine serum albumin nanoparticles to bind to a surface-modified E-selectin peptide by coupling the sulfhydryl group on the E-selectin-binding peptide with the amino group on the surface of bovine serum albumin; these nanoparticles could be loaded with the anti-inflammatory drug dexamethasone and specifically targeted to activated lung endothelial cells (Fig. 5).

#### P-selectin

P-selectin (CD62P) is a member of the selectin family of cell adhesion molecules that is expressed on stimulated endothelial cells and activated platelets [71]. P Selectins act primarily in adhesion between leukocytes, platelets, and endothelial cells and promote leukocyte migration, adhesion to the endothelial surface of blood vessels, and thus leukocyte infiltration into tissues [72, 73]. N. Dai et al. [74] proposed the use of the galactolactan polysaccharide from the traditional Chinese herb Viola diffusa to alleviate lipopolysaccharide (LPS)-induced ALI by inhibiting the activation of P-selectin-dependent adherence and recruitment of neutrophils to the endothelium.

C. Liu et al. [75] developed a self-assembled nanoparticle (NP) called DIP-FU-PPy NP, which consists of polypyrrole (PPy), dipyrrole (DIP), and P-selectin-targeted violet quinoa (FU). These NPs are designed to be delivered directly to the lesion area with drug loading capacity, thrombus site targeting ability, and near-infrared (NIR) photothermal effects, all of which could effectively prevent thrombus recurrence. Chia-Hung Liu’s study is important for preventing pulmonary thrombosis during ALI when the coagulation function of pulmonary microvessels is abnormal. Thus, P-selectin could be a reliable target for the treatment of ALI.

#### Intercellular adhesion molecule-1 (ICAM-1)

ICAM-1 is expressed predominantly on endothelial cells, mediates leukocyte-to-leukocyte and leukocyte-to-cell adhesion, and is critical for leukocyte migration and activation [76]. ICAM-1 expression is low in resting endothelial cells and is upregulated during ALI, which promotes leukocyte adhesion to endothelial cells, allowing leukocytes to leak out into the tissue interstitium and exacerbating inflammatory responses and tissue damage [77]. In addition, ICAM-1 is involved in the migration and infiltration of neutrophils and macrophages into lung tissues, further exacerbating lung injury.^[67]^

Treatment of ALI by targeting ICAM-1 has been reported previously. S. Jiang [78] designed a ternary nanoparticle (ICAM-NLC/Pro/Ang) containing anti-cellular ICAM-1, which could be targeted for the treatment of ALI and significantly reduced TNF-α and IL-6 levels in the lungs of mice. Cytotoxicity assays showed that the nanocarrier had low cytotoxicity. Thus, the nanosystem not only is biocompatible but also has good lung therapeutic efficacy. This further confirmed the feasibility of the targeting site.

#### Rab26

Disruption of the endothelial barrier function of the pulmonary microvasculature is the main pathophysiologic mechanism of ALI and is closely related to fluid accumulation and leukocyte extravasation into the alveolar luminal space [79]. Rab26 can regulate vesicle transport from the Golgi to the membrane by interacting with the adrenergic receptor α2A (ADRA2A) [80]. Maintenance of endothelial cell adhesion junction stability mediated by ADRA2A prevents neutrophil extravasation during the inflammatory response. RAB26 overexpression promotes the interaction of SRC with the autophagy protein light chain 3-II (LC3-II) and facilitates the degradation of phosphorylated SRC proteins. These findings suggest that RAB26 plays a protective role in regulating EC permeability, mainly by targeting the autophagic degradation of phosphorylated SRC proteins and maintaining the expression of the adhesion junction protein VE-cadherin in a dephosphorylated state, thus protecting the stability of adhesion junctions [81].

H. Li [82] designed self-assembled Rab26 siRNA nanoparticles loaded with the DNA Y-motif (siRab26-DYM), which could exacerbate apoptosis in HPMECs by activating the Toll-like receptor 4 (TLR4) signaling pathway. Overexpression of Rab26 by Rab26 adenovirus partially inactivated the TLR4 signaling pathway and inhibited apoptosis. Rab26 may be a potential therapeutic target for the treatment of vascular diseases related to endothelial barrier function.

#### NE

Neutrophils are important effector cells in the pathogenesis of ALI, and their activation and migration are closely related to the severity of ALI [83]. Activated neutrophils can produce many cytotoxic substances, including granzymes, reactive oxygen species (ROS), reactive lipids, various proinflammatory cytokines, and neutrophil extracellular traps; these substances can lead to cellular damage in lung tissues and are essential for ALI development [84].

#### Neutrophil extracellular traps (NETs)

NETs are composed mainly of free DNA, histones, neutrophil elastase and myeloperoxidase (MPO) [85]. NETs protect the host by trapping and killing pathogens. However, excessive NET formation can stimulate and synergize with other cells, leading to amplification of the cascade response to sterile inflammation, and these pathological processes can cause damage to the organism [86, 87].

**Fig. 6:**
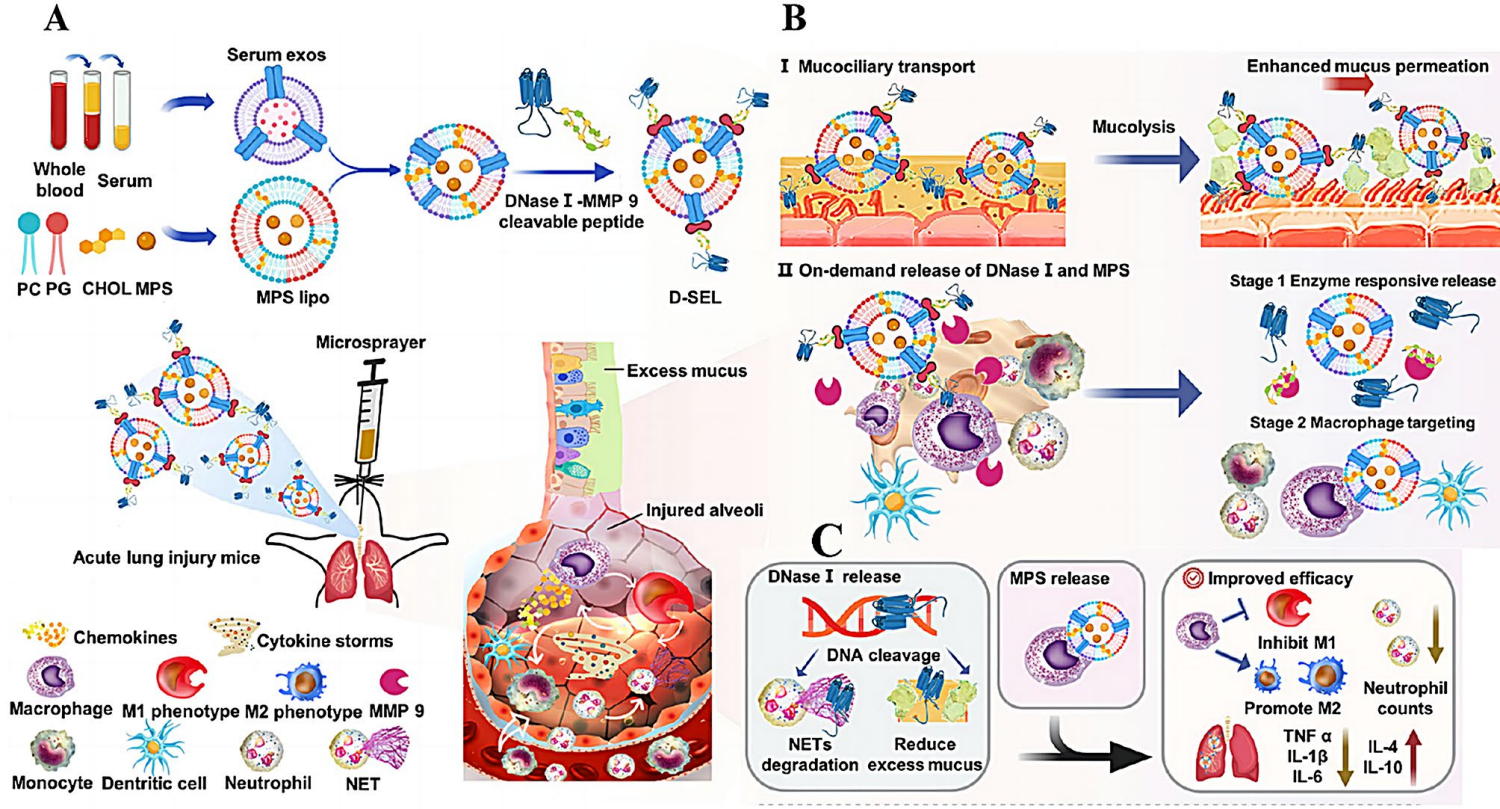
Inhalation of MPS/D-SEL promoted dysregulated NET degradation in ALI mice. **(A)** Illustration of the MPS/D-SEL construction. **(B)** MPS/D-SELs displayed superior mucus penetration ability and were retained in the alveoli after inhalation. DNase I was then released from the nanocarrier first after responding to MMP-9, resulting in inner SEL core exposure, which precisely delivered MPS into macrophages to promote M2 macrophage polarization. **(C)** The synergistic anti-inflammatory effects of MPS/D-SEL include the degradation of dysregulated NETs, suppression of the mucus-blocking microenvironment, and promotion of M2 macrophage polarization. Adapted with permission from ref [88].,copyright 2023, American Chemical Society

C. Liu [88] designed an inhalable drug-releasing nanoplatform (D-SEL), which was then constructed by adding methylprednisolone sodium succinate (MPS). The main mechanism of action of the drug is that MMP-9 in the D-SEL platform enables the release of DNase I from the nanocarrier, leading to SEL-related core exposure, which accurately delivers MPS to macrophages for action. The sustained release of DNase I degrades dysregulated NETs and inhibits neutrophil activation and mucus-clogged microenvironments, thereby increasing the polarization efficiency of M2 macrophages. (Fig. 6). Therefore, degrading NETs could be a reliable target for the treatment of ALI.

#### Iron-induced death of NE cells

Iron-related modifiable cell death, which is characterized mainly by the iron-dependent accumulation of lipid peroxides, has been proposed in recent years [89]. Intracellular lipid peroxides are produced via lipid reactive oxygen species that undergo the iron-Fenton reaction, oxidative esterification of polyunsaturated fatty acids, and iron-catalyzed lipid autoxidation [90, 91]. The deposition of lipid peroxides induces the development of iron death, and the onset of lung injury is accompanied by a large amount of neutrophil infiltration. S. Deng [92] reported that quercetin could ameliorate lung injury by reducing reactive oxygen species production and proinflammatory cytokine release and by reducing neutrophil iron release. Therefore, targeting neutrophil iron death has an important role in regulating ALI.

#### Cellular pyroptosis of NE cells

Cellular pyroptosis can occur through inflammation-associated caspase-1 or caspase-4/caspase-5/caspase-l1-dependent programmed cell death [93]. Inflammatory vesicles trigger neutrophil pyroptosis, and neutrophils swell and undergo membrane lysis, releasing large amounts of immunomodulatory cytokines and chemokines, such as interleukin-10 (IL-10), interleukin-13 (IL-13), interleukin-8 (IL-8), macrophage inflammatory protein-1, MPO, histone G, and other granulomatous enzymes, which lead to increased lung load and, consequently, more severe inflammation and ALI [94–96]. After neutrophil membrane lysis, intracellular pathogens are not directly released to the outside of the cell but can form pore-induced intracellular traps, which coordinate the innate immune response through complement and scavenger receptors to promote neutrophil recruitment to release reactive oxygen species or secondary phagocytosis to kill the pathogen [97, 98]. J. Kang [99] found that melatonin was able to prevent the activation of nuclear factor erythroid 2-related factor (Nrf-2) signaling, which in turn drove the antioxidant pathway, including a significant increase in the expression of Mn-SOD, NQO1, HO-1, Nrf2, and catalase in vivo and in vitro. Moreover, melatonin inhibited the overproduction of ROS and MDA, the expression of inducible nitric oxide synthase (iNOS), and the expression and release of TNF-α and IL-1β. In addition, melatonin inhibited LPS-induced ALI-related death by reversing the increase in the expression of NOD-like receptor thermal protein domain associated protein 3 (NLRP3), Caspase-1, IL-1β, IL-18, and N-gasdermin-D (N-GSDMD). Thus, modulating neutrophil pyroptosis could provide new ideas for the prevention and treatment of ALI.

#### Apoptosis of NE cells

Neutrophil apoptosis is a complex process regulated by multiple genes and involves numerous cytokines and multiple signaling pathways [100]. At the onset of ALI, IL-8, TNF-α, IL-6, and granulocyte colony-stimulating factor are activated, and neutrophils are recruited into the interstitium and alveoli, leading to progressive loss of lung function. Delayed neutrophil apoptosis can even lead to the formation of NETs, which interact with macrophages and thus contribute to ALI [101].

The B-cell lymphoma-2 (Bcl-2) inhibitor venetoclax promotes neutrophil apoptosis and improves the clinical prognosis of ALI patients; however, the water insolubility of free venetoclax hinders its pulmonary delivery. R. Su [102] designed an amphiphilic poly(ethylene glycol)-modified poly(alpha-lipoic acid) nanoparticle, which enables venetoclax to remain in lung tissue for a longer period of time and shows better efficacy against ALI by increasing neutrophil apoptosis in vivo. Therefore, timely enhancement of normal neutrophil apoptosis is an important approach in the treatment of inflammatory diseases.

#### Macrophages (AMs)

**Fig. 7:**
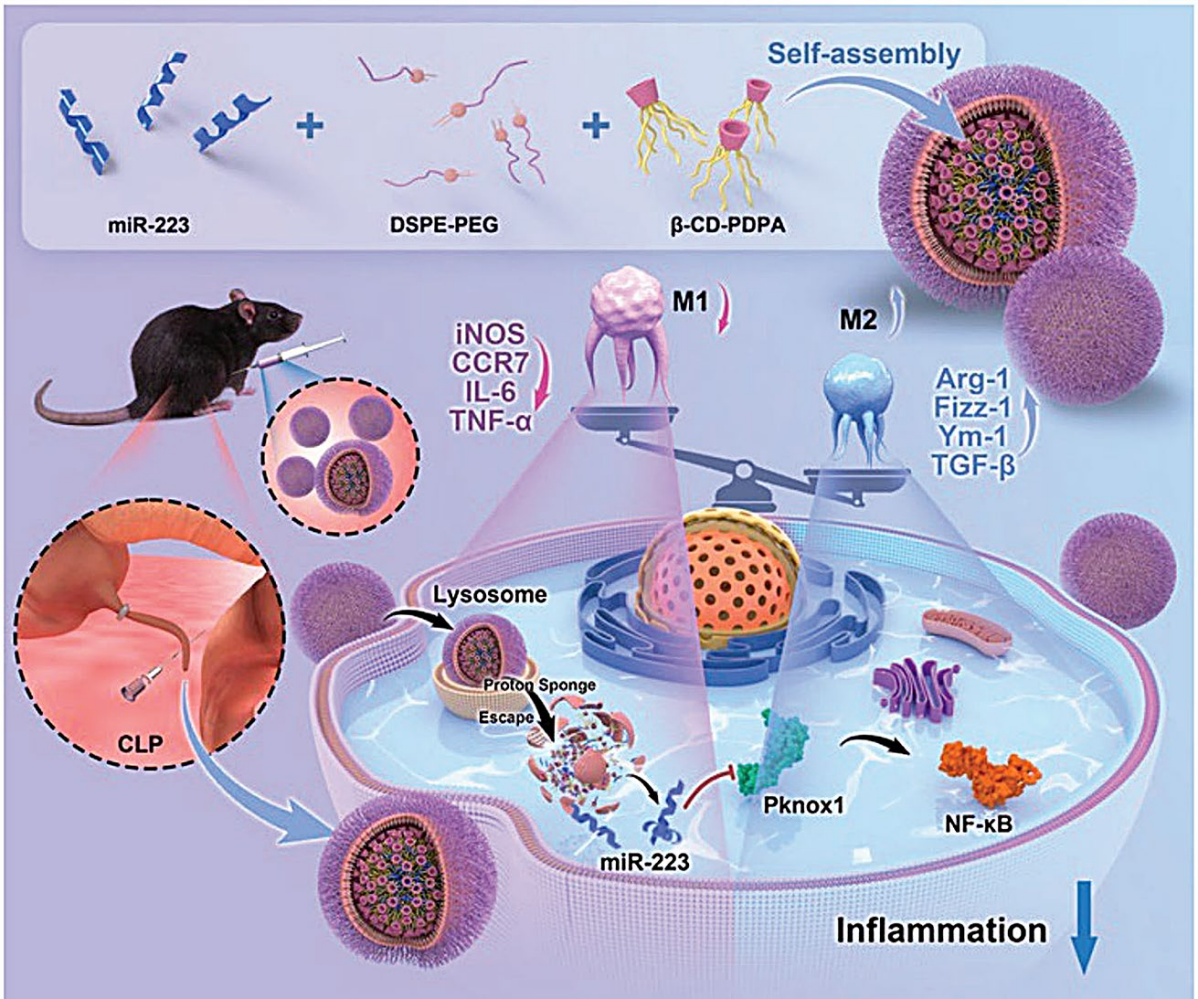
NPs/miR-223 switches the M1 phenotype to the M2 phenotypein vitro. Schematic representation of the design, synthesis, and delivery of NPs/miR-223 for sepsis treatment. Mechanistically, NPs/miR-223 switched M1 macrophages to the M2 phenotype by targeting Pknox1 and inhibiting the activation of the NF-κB signaling pathway. adapted with permission from ref [108].,Copyright 2023, Wiley-VCH.

Pulmonary AMs are found in the interstitium and alveoli and are recruited in large numbers to lung tissue in response to inflammatory stimuli [103]. They possess a complex set of mechanisms for defense against invasion by foreign bodies and pathogens. When macrophages are stimulated, resting macrophages (M0) are classically activated instead of differentiating into M1-type macrophages and M2-type macrophages [104, 105]. M1 macrophages release a variety of potent proinflammatory cytokines, including TNF-α, IL-6 and IL-1β. Lung macrophages are the main coordinating cells involved in the termination and regression of lung inflammation, and modulation of AMs has been found to attenuate lung injury by attenuating neutrophil accumulation and reducing proinflammatory cytokines; however, much work still needs to be done to continue investigating the specific signaling pathways and cellular mechanisms involved in inflammation [106, 107]. N. Ding [108] developed a pH-responsive nanodelivery system consisting of β-cyclodextrin-poly(2-(diisopropylamino)ethylmethacrylate)/distearoylphosphatidylethanolamine-polyethyleneglycol (β-CD-PDPA/DSPE-PEG) for targeting M1 macrophages and encapsulating miR-223 in NPs for ALI therapy (Fig. 7). D. Sun [109] constructed two anti-inflammatory N-acetylcysteine (NAC)-loaded macrophage-targeting apoptotic cell-inspired phosphatidylserine (PS)-containing nanoliposomes (PSLipos). The softer PSLipos-L-NAC inhibited the inflammatory response more effectively by binding to the macrophage surface and prolonging the duration of action of the targeted macrophages, which in turn accelerated wound healing in the inflamed lung epithelium. In a mouse model of ALI, the administration of PSLipos-L-NAC through the lungs significantly reduced the inflammatory response of M1-like macrophages in lung tissue and promoted lung injury repair. Therefore, effective promotion of macrophage polarization is an important target for the treatment of ALI.

#### Mitochondria (mt)

Abnormal mitochondrial and mitochondrial DNA (mtDNA) levels are associated with the development of ALI/ARDS, and plasma mtDNA levels are expected to be biomarkers for the clinical diagnosis and assessment of the severity of lung injury. Mechanistically, mtDNA released from damaged mitochondria and its oxidized form play crucial roles in the inflammatory response and histopathological changes in the lungs [110]. Mitochondria play pivotal roles in the cell as an energy supply system. When inflammation occurs in the lungs, neutrophils are activated and release reactive oxygen species. The main organelles attacked by reactive oxygen species are mitochondria, and the balance between mitochondrial fusion and fission is disrupted and dysfunctional due to the occurrence of massive oxidative stress [111]. Mitochondrial damage-associated molecular patterns (MTDs) are a type of damage-associated molecular pattern (DAMP) released by mitochondrial rupture that induces inflammation and is involved in the pathogenesis of ALI.

**Fig. 8:**
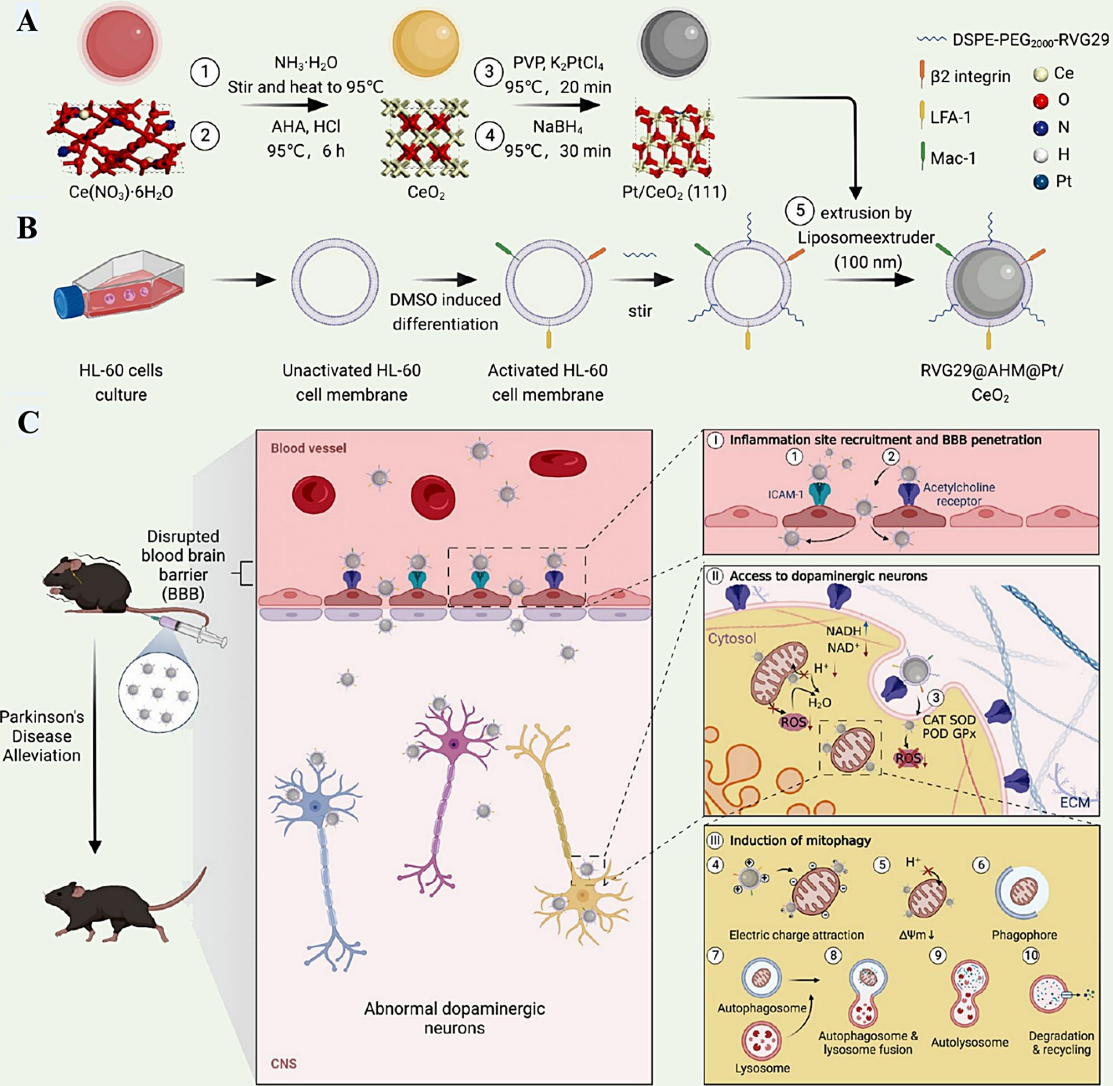
Exploration of the mechanism of mitophagy promotion by the single-atom catalyst Pt/CeO2. **(A)** Synthesis process of single-atom catalysts (SACs) Pt/CeO2. **(B)** Extraction and activation of neutrophil-like (HL-60) cell membranes, modification of the rabies virus glycoprotein (RVG29) peptide on HL-60 cell membranes, and preparation of SACs with a core − shell structure. **(C)**In vivo mechanism of action of core − shell-structured SACs. adapted with permission from ref [113].,copyright 2023, the American Chemical Society

W. Peng [112] first constructed an MTD-induced mouse ALI model and used rapamycin to regulate autophagy; the team found that with increasing autophagy, the secretion of inflammatory factors decreased, and the expression of NLRP3 inflammation-associated proteins decreased, which effectively attenuated lung injury. B. Li [113] designed a single-atom catalyst (SAC), Pt/CeO2, which not only promotes ROS catabolism by interfering with the α-glycerophosphate shuttle pathway and the malate-aspartate shuttle pathway but also induces a depolarization of the mitochondrial membrane potential, causing the mitochondria to clean themselves and eliminate the source of ROS generation (Fig. 8). Therefore, promoting mitochondrial autophagy, which can effectively remove ROS, is an effective target for the treatment of ALI.

#### Regulation of immune metabolism

Immune metabolism regulation focuses on the regulation of immune cells when the body is damaged. In addition to self-recognition, the immune system also regulates the internal environment after injury, including damage caused by hypoxia, toxins, infection, and mechanical damage. When injury occurs, immune cells can respond quickly and transmit local and systemic signals to cytokines or antibodies to trigger an adaptive response to injury [114]. The immune system further regulates and monitors the repair of damage, ultimately reestablishing immune homeostasis [115, 116]. Both natural and acquired immunity are involved in different stages of ALI. In innate immunity, the precise immune regulation of macrophages is essential for maintaining the homeostasis of lung inflammation [117]. For adaptive immunity, naïve T and B-cell metabolic programs are dynamically regulated at the onset of injury [118].

#### Dendritic cells (DCs)

**Fig. 9:**
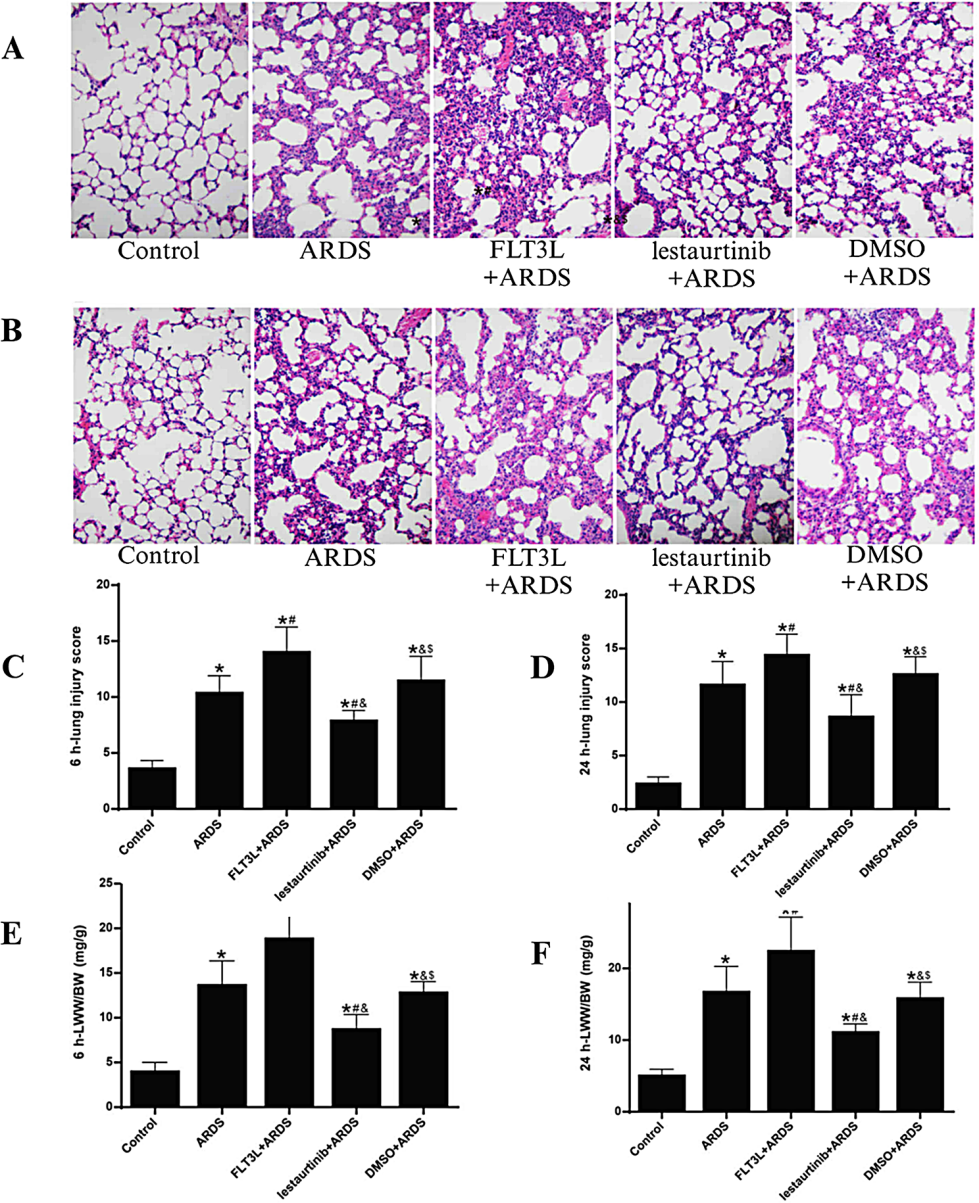
Effect of cDC manipulation on lung edema and lung injury. **(A)** Hematoxylin and eosin staining (magnification, ×200) of lung tissues at 6 h and **(B)** at 24 h post-LPS challenge. **(C)** Lung injury score at 6 h and **(D)** at 24 h post-LPS challenge. **(E)** LWW/BW at 6 h and **(F)** at 24 h post-LPS challenge. The lung injury score is expressed as an arbitrary mean. The data are presented as the mean ± standard deviation (*n* = 6). adapted with permission from ref [120].,copyright 2019, Spandidos

Inflammatory diseases of the lung are closely related to T-cell-mediated immune responses, and the main pathway is the processing of antigens by DCs, which act by presenting information to specific T cells [119]. L. Li [120] investigated the role of lung cDCs in the regulation of LPS-induced ALI (Fig. 9). ALI was first induced using LPS pretreatment for five days, and the levels of each inflammatory factor, as well as the levels of the transcription factors T-box-expressed-in-T-cell (T-bet) and GATA-binding protein 3, were measured to assess the balance of the Th1/Th2 response. Fms-like tyrosine kinase 3-ligand (FLT3L) or letrutinib (a specific activator and inhibitor of FLT3 signaling, respectively) was then injected intratracheally. The aggregation and maturation of lung cDCs peaked at 6 h after LPS stimulation; however, letrutinib inhibited the aggregation and maturation of lung cDCs and ameliorated acute lung inflammation. Lung cDCs may modulate acute lung injury by regulating neutrophil infiltration and the balance of Th1/Th2 responses. Z. Lu [121] showed that the recruitment and maturation of lung DCs is an important process in the early stage of ALI and that mesenchymal stem/stromal cells (MSCs) attenuate LPS-induced ALI by activating Notch signaling to induce the production of DCregs.

#### NK

cells lack T- and B-cell-specific markers such as TCR and mIg on their surface, and these cells are not dependent on antigenic stimulation and can spontaneously lyse a wide range of tumor cells and virus-infected cells [122, 123]. ALI is a major causative factor of paraquat-induced mortality, and in a model of acute paraquat intoxication, depletion of NK cells attenuated paraquat-induced lung injury by attenuating macrophage and neutrophil infiltration in the lungs and reversing paraquat-induced macrophage polarization to the proinflammatory M1 phenotype [124]. Therefore, reducing NK production reduces lung damage and is a reliable therapeutic target.

#### T cells

Cellular immune responses mediated by T cells that recognize and control pathogens are important components of adaptive immunity, and effective T-cell responses to infection depend on coordinated metabolic reprogramming among various immune cells [125, 126]. Depending on their origin, regulatory T cells (Tregs) can be categorized into natural Tregs (nTregs) and induced Tregs (iTregs), which are derived from thymocytes and naïve T helper cells, respectively [127]. Compared with iTregs, which have more regulatory functions and express CD4 + CD25 + Foxp3 + surface biomarkers, nTregs are more common. ALI leads to the exclusive expansion of CD4 + Tregs, which is dependent on self-antigen recognition and interleukin-2 (IL-2). In contrast, conventional CD4 + T cells with the same autoantigen specificity remain unresponsive even after Treg ablation. Thus, the self-antigen-specific CD4 + T-cell group may exert a regulatory function during acute tissue injury, limiting the potential for further injury and autoimmunity [128]. A common complication of sepsis is ALI. Tregs and T helper 17 (Th17) cells constitute the CD4 + T-cell subset and strongly influence inflammation during ALI. L. Chen [129] investigated the effects of the immunomodulatory drug berberine (BBR) on the inflammatory response and immune status of septic mice and reported that BBR modulated Treg/Th17 homeostasis and downregulated NF-κB signaling to alleviate ALI in septic mice. Therefore, the regulation of T-cell homeostasis is extremely important in the treatment of ALI.

#### B cells

B cells are categorized as B1 cells, B2 cells (including follicular cells and MZ cells), or regulatory B cells [130]. IL-10 is a potent anti-inflammatory factor that is produced by B cells. Z. Sun [131] reported that blockade of IL-10 signaling aggravated lung injury by inducing the production of proinflammatory factors and neutrophil recruitment to the lungs. B-cell-derived IL-10 inhibits macrophage activation and recruitment and downregulates the production of the chemokine KC, which recruits neutrophils to the lungs, and exogenously supplied IL-10 promotes recovery from LPS-induced ALI [132]. Therefore, the production of higher levels of IL-10 by regulating B cells is valuable in the treatment of ALI.

## Types of nanomaterials targeted to treat ALI

The effectiveness of enzyme inhibitors and hydrophobic drugs in treating acute lung injury is limited due to their poor stability [133]. In recent years, nanomedicines have been gradually applied to the treatment of acute lung injury, which can not only improve the targeting ability of drugs but also improve the biosafety of drugs [134]. The more common targeting nanomaterials are mainly polymer-based NPs, liposome-based NPs, peptide-based NPs, inorganic-based NPs, and nanoparticles camouflaged by cell membranes or associated microvesicles.(Table 1).

**Table 1:** Targets for the treatment of ALI after burn

Targeing cells	Targeting moiety	Types of NPs	Drug	Principles of treatment	Ref.
Passive targeting	Sites of Lung Injury	Polymer-based	Crocin	Reducing levels of inflammatory cytokines and modulating oxidative stress.	[64]
ECs	E-selectin	Peptide-based	BSA	Reducing levels of inflammatory cytokines	[70]
	P-selectin	Polymer-based	Fucoidan	Targeted accumulation at thrombus sites	[75]
	ICAM-1	Liposome-based	Angiopoietin-1	Reduces TNF-α and IL-6 levels	[78]
	Rab26	Camouflaged NPs	Rab26 siRNA	Activation of the TLR4 signaling pathway	[82]]
NEs	NETs	Liposome-based	MPSS	Degradation NETs, promotes M2 polarization	[88]
	iron death	-	Quercetin	Inhibition of iron death	[92]
	cellular pyroptosis	-	Melatonin	Inhibited the expression of iNOS	[99]
	apoptosis	Polymer-based	Venetoclax	Promote apoptosis	[102]
AMs	M1	Liposome-based	N-acetylcysteine	Prolonged targeting of AMs and reduced M1	[109]
	M2	Camouflaged NPs	MSC	Adaptive tuning of the Nrf2 defense system	[105]
	PrCR	Camouflaged NPs	Doxorubicin	Starting the PrCR too early	[110]
Mts	membrane potential	Inorganic-based	Pt/CeO2	Induction of mitochondrial membrane potential (Δψm) depolarization	[113]
Immune metablism	DCs	Camouflaged NPs	MSC	Activation of Notch signaling	[121]
	NK	-	-	Suppression of M1 polarization	[124]
	T-cell	-	Berberine	Regulation of Treg/Th17 homeostasis and downregulation of NF-κB signaling	[129]
	B-cell	Camouflaged NPs	MSC	Promotes IL-10 production by B cells	[131]

### Liposome-based NPs

The ability of liposomal nanomaterials to target the lungs is largely based on their unique design and biological properties, which depend on their physicochemical properties, surface modifications, and mode of delivery [135, 136]. The following are several of the key factors and mechanisms: (1) Size and surface properties: the size and surface properties of liposomes can be precisely controlled to facilitate their accumulation in the lungs. For example, liposomes of a particular size are more likely to be intercepted by the capillary network in the lungs. (2) Surface modifications: Liposomal surfaces can be modified to increase their affinity for specific lung cell receptors or tissues. These modifications may include specific ligands, antibodies, or other molecules that specifically recognize and bind to particular cells or tissues in the lungs [137]. (3) Inhalation route: liposomes can be delivered directly to the lungs by inhalation. This route brings the drug directly to the lungs and reduces the amount of drug circulating systemically, thus reducing systemic side effects [138, 139]. (4) Capitalizing on pathology: ALI is often accompanied by inflammation and increased vascular permeability. Liposomes can take advantage of these pathological changes to penetrate injured lung tissue more easily. (5) Prolonging circulation time: By adjusting the composition of liposomes, they can be made to circulate in the bloodstream for a longer period of time, thereby increasing their chances of reaching and accumulating in the lungs. (6) Stability of liposomes: liposomes are designed to ensure that they remain stable until they reach their destination, preventing premature drug release [140]. These properties make liposomes effective vehicles for the treatment of lung diseases.

Lipid nanoparticles (LNPs) are widely used in the clinic for mRNA delivery, but their application is limited by the formation of apolipoprotein E (ApoE)-rich protein crowns, which can accumulate in large quantities in the liver. S. Dilliard [141] developed selective organ targeting (SORT) LNPs containing a complementary component (called the SORT molecule) for the delivery of tissue-specific mRNA to the liver, spleen and lungs of mice. Changes in the chemical structure of the tails and heads of the lipid alkyl groups affect the efficacy and specificity of mRNA delivery to the lungs. In a study of LPS-induced ALI in pigs, Fisher. AB ingeniously designed LNPs encapsulating a nine amino acid peptide [peroxiredoxin 6 PLA(2) inhibitory peptide-2 (PIP-2)], which is derived from the lung surface-active protein A. The peptide is able to inhibit peroxiredoxin 6 activity and the subsequent activation of NOX1,2, thereby inhibiting reactive oxygen species (ROS) production. The presence of liposomes in the nanoparticles not only controlled the release of PIP-2 but also targeted the lungs. In in vivo experiments, bronchoalveolar lavage fluid from PIP-2-treated pigs showed significant reductions in the levels of total proteins, cytokines (TNF-α, IL-6, and IL-1β), and myeloperoxidase, which verified the advantages of peptide-loaded lipid nanoparticles in the treatment of acute lung injury [142].

In conclusion, LNPs are a reliable drug delivery system, and their surface can be modified as needed to target the lungs more efficiently and slow acute lung injury.

### Polymer-based NPs

Polymer nanoparticles are prepared using natural polymers such as dextran, chitosan, cyclodextrins or synthetic polymers, which allows the fabrication of polymer nanoparticles with different compositions and structures. As a drug delivery carrier, polymer-based NPs are easy to synthesize and characterize. It has the advantages of increased biocompatibility, biodegradability, strong bioadhesion, nonimmunogenicity, nontoxicity and water solubility. Both in vitro cellular and animal experiments confirmed that it can be effectively encapsulated and that it can effectively release drugs. With its good bioadhesive property, chitosan can achieve cross-barrier drug delivery (e.g., blood‒brain barrier), and to a certain extent, it can achieve targeted drug delivery [61]. Glucocorticosteroids, such as methylprednisolone sodium succinate (MPSS), can be effective in the treatment of ALI. However, frequent and prolonged administration of high doses of glucocorticosteroids can lead to hormone dependence and severe side effects. Y. Ding [143] combined nanoparticles with erythrocytes for targeted delivery of MPSS to the lungs. The preparation of chitosan nanoparticles loaded with MPSS (MPSS-CSNPs) and adsorbed on the surface of erythrocytes through noncovalent interactions (RBC-MPSS-CSNPs) showed satisfactory efficiency in lung delivery and could be used against ALI, which avoids damage to other organs.

However, some of the polymer-based NPs may be toxic to normal cells due to their positive charge. Therefore, Sun HX designed a phosphorus dendritic polymer capped with anionic phosphite, named AK-137, which binds to the protein drug of interest mainly through physical interactions. Fibronectin is extremely important in organisms, and the authors loaded fibronectin on AK-137 and synthesized AK-137@FN nanocomplexes, which showed potent therapeutic effects in an acute lung injury model [144]. Therefore, polymer-based NPs are valuable as drug delivery vehicles for the treatment of ALI.

### Peptide-based nanoparticles

Among the various ligands, peptides have received great attention due to their biocompatibility, fine-grained functionality, and stability relative to protein/protein structural domains [145]. Some polypeptides contain specific amino acid sequences that specifically recognize and bind to surface receptors or molecules on lung cells. By selecting peptide sequences with high affinity, nanoparticles can target lung tissue more effectively than can other agents [146, 147]. Peptides are biodegradable, which means that they can be broken down and absorbed naturally by the body after they have accomplished their drug delivery task, reducing the risk of long-term toxicity [148].

B. Ouyang [149] developed an albumin-based nanopreparation for the active delivery of furfuracin (FMN) to improve the treatment of lung injury and fibrosis. The obtained FMN@BSA NPs efficiently accumulated at damaged lesions due to leakage from the vascular system and the affinity between albumin and the overexpressed SPARC proteins. By blocking the macrophage pyroptosis process involving NLRP3 inflammatory vesicles, FMN@BSA NPs significantly improved lung function and prolonged animal survival in a bleomycin (BLM)-induced model of ALI and fibrosis without significant side effects. In conclusion, by utilizing specific amino acid sequences, peptides can specifically bind to receptors on damaged lung cells, allowing the nanomaterials to function more efficiently, and the peptides are degradable, reducing biotoxicity.

### Inorganic-based NPs

The mechanisms by which inorganic nanomaterials target the lungs usually involve combining their physicochemical properties with biological strategies, and their surfaces can be modified to increase their targeting properties [150]. This may include the attachment of specific ligands, antibodies, or other molecules that bind specifically to receptors or other biomarkers in lung cells [151]. Smaller nanoparticles may be more likely to cross biological barriers and enter lung tissue. Inorganic nanomaterials can be engineered to alter their properties (e.g., solubility, magnetism, or fluorescence) under specific physiological or pathological conditions, thereby enhancing their targeting and therapeutic efficacy. Although inorganic nanomaterials have potential for targeted delivery, their biocompatibility and potential toxicity are important considerations [152].

H. Li [153] designed a γ-cyclodextrin metal-organic framework (CD-MOF) with a respirable particle size, cubic morphology, and good aerodynamic properties for targeted pulmonary drug delivery via a dry powder inhaler. The team used uniformly sized γ-cyclodextrin metal-organic skeletons (CD-MOFs) as inhalable carriers to deliver the natural product salbutamol (PAE) for the treatment of ALI, and intratracheal administration of PAE-CD-MOF powder resulted in immediate absorption and higher plasma concentrations than did pure salbutamol inhalation and oral administration. Histopathologic studies have shown significant reductions in therapeutic efficacy and inflammatory factor levels following the inhalation of PAE-CD-MOF powder. In addition to metallic materials, silica nanoparticles (MSNs) can also serve as promising nanocarriers for pulmonary drug delivery [154]. In conclusion, inorganic nanomaterials with smaller particle sizes, easier access to the lungs through the biological barrier to perform their functions, and a three-dimensional structure to carry more drugs with higher solubility are highly valuable for the treatment of acute lung injury.

### Nanoparticles camouflaged by intrinsic cell membranes or associated microvesicles (camouflaged NPs)

The strategy of blocking nanoparticles by intrinsic cell membranes or associated microvesicles for targeting the lungs relies primarily on their ability to mimic cellular or cellular components and to interact with specific cells or tissues in the lungs [155]. The use of cell membranes or microvesicles to encapsulate nanoparticles reduces their recognition and removal by the immune system, as these membranes are derived from the body’s own cells and are naturally biocompatible. Cell membranes or microvesicles may contain specific proteins or receptors that can bind to specific markers in lung cells, thus improving lung targeting [156]. Camouflaged nanoparticles can cross biological barriers, such as the blood‒air barrier, more efficiently, thereby increasing the efficiency of the drug in reaching the lungs. Due to the high biocompatibility of camouflaged nanoparticles, they may reduce side effects on nontargeted organs, especially by reducing accumulation in the liver and spleen [157].

**Fig. 10:**
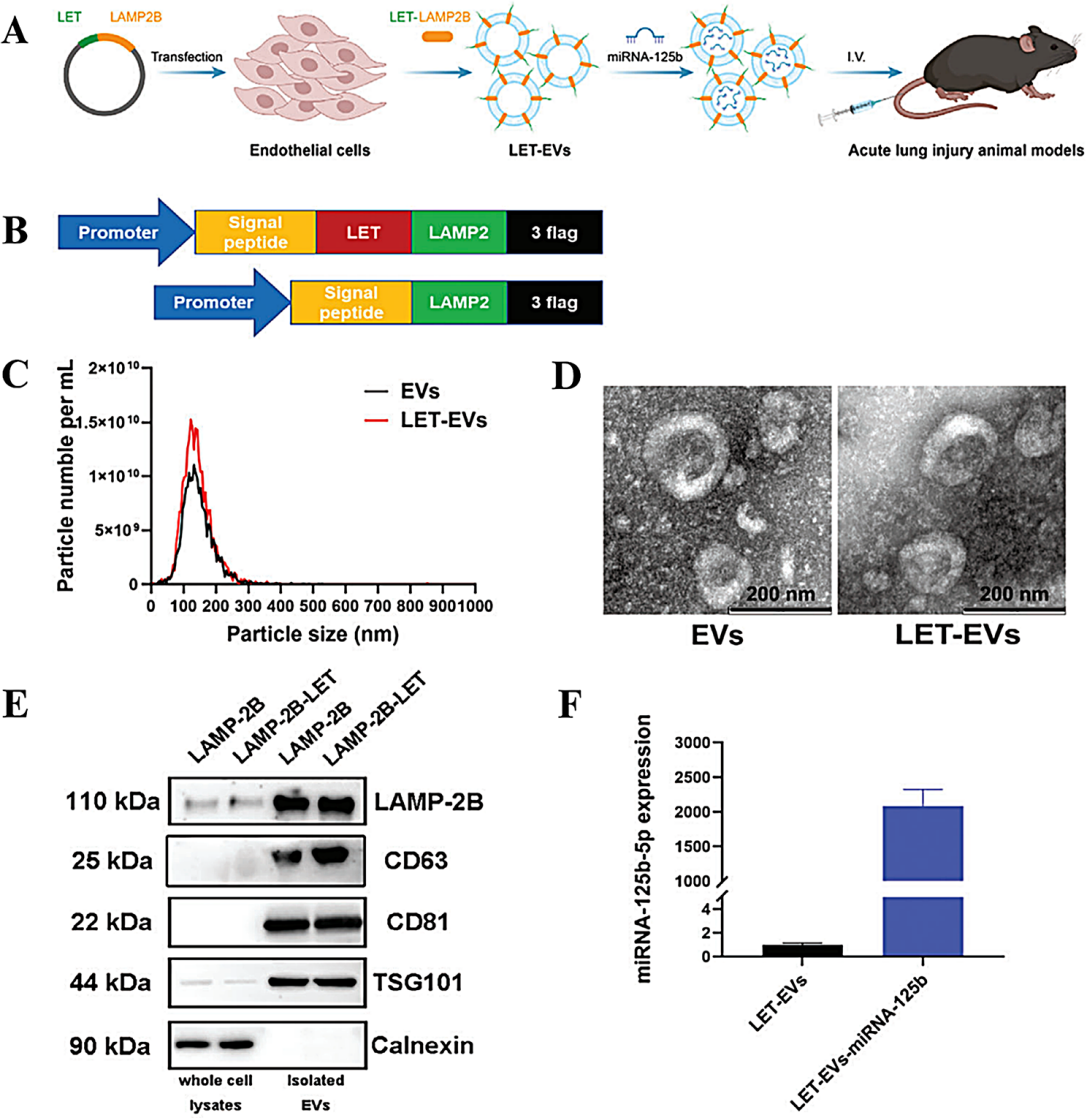
Design and characterization of LET-EVs-miRNA-125b. **(A)** Schematic diagram of the preparation of LET-EVs-miRNA125b. **(B)** Schematic of the construction and production of the LAMP-2B-LET fusion protein plasmid. **(C)** Size distribution and particle distribution of EVs and LET-EVs, as measured via NTA. **(D)** The morphology of EVs and LET-EVs was a typical cup-shaped structure, as detected by TEM. **(E)** EV-positive (LAMP-2B, CD63, CD81, and TSG101) and -negative (calnexin) markers were detected in LAMP-2B-EVs, LAMP-2B-LET-EVs, and their donor cells by western blotting. **(F)** The loading efficiency of miRNA-125b-5p was detected using qRT‒PCR to compare the relative expression of miRNA-125b-5p between LET-EVs and LET-EVs-miRNA-125b (*n* = 3). adapted with permission from ref [158].,Copyright 2023, Wiley-VCH

Z. Gu [158] engineered extracellular vesicles (eEVs) of endothelial origin by delivering microRNA-125b-5p (miRNA-125b) to lung tissues and protecting endothelial barrier integrity. To improve the efficacy of these EVs, miRNA-125b was added to the EVs to construct LET-EVs-miRNA-125b. The results showed that LET-EVs-miRNA-125b had the most significant therapeutic effect on ALI compared with EVs, miRNA-125b and EVs-miRNA-125b (Fig. 10). In addition, the application of MSC membranes has gradually developed. Jin. H designed a biomimetic nanoparticle in which MSC membranes (CMs) were skillfully encapsulated with naringenin nanoparticles (Nar-NPs) by the hand extrusion method to obtain biomimetic CM@Nar-NPs. NPs are highly biocompatible and biosafe due to the presence of CMs and can target inflammatory sites in the lungs and modulate macrophages, thus exerting anti-inflammatory effects [159].

In summary, by using cell membranes or associated vesicles to block nanomaterials, it is possible to reduce the recognition and clearance of drugs by the immune system, cross the biological barrier to the lungs more efficiently to perform their functions, and reduce side effects.

## Conclusions and future perspectives

Nanomedicines currently used for the treatment of postburn ALI have shown promising results, but their delivery efficiency is not high. Understanding the pathogenesis of ALI is important for targeted therapy. An increase in drug delivery efficiency would not only maximize the therapeutic effect but also reduce the required dose, thereby reducing the side effects of ALI treatment. In this study, the mechanism of ALI after burn injury is discussed, therapeutic targets are summarized according to their pathophysiological characteristics, and nanomaterials are classified. However, please note that related research is still in its infancy, and key issues remain to be resolved.

First, it is worth noting that specific targeting also has some potential risks, such as unwanted immune responses. A breakthrough in this area would greatly facilitate the development of this bionic approach. The use of autologous cells such as macrophages, neutrophils, and MSCs to transport nanomedicines may constitute a future research direction [160]. They are effective in preventing recognition and removal by organisms. It is possible to genetically modify isolated cells to reduce immunogenicity when performing in vitro experiments. However, the extraction of autologous cell membranes is still in the preliminary stage, so how to extract cell membranes quickly and effectively will be important for targeting ALI. The adequacy of the number of autologous cells should be considered. The acquisition of cell membranes and extracellular vesicles requires extraction from autologous cells. At this point, whether these cells can be expanded in vitro is unknown. When these issues are resolved, autologous cellular transport of nanomedicines is highly valuable in the targeted treatment of ALI.

Second, the mechanism of acute lung injury must be further investigated. Nanomaterials based on neutrophils, mitochondria, endothelial cells, and other targeted therapies have been widely investigated; however, the effects of immunometabolic modulation, an important part of acute lung injury treatment, on nanomaterials targeting T cells, B cells, and other immunometabolic therapies have rarely been reported. The different mechanisms of ALI should be fully investigated, and novel nanomaterials should be developed to further guide the treatment of ALI.

Third, the mode of administration should be improved. Currently, the more common modes of drug delivery include powder inhalation, intravenous injection, and tracheal drip, which have problems such as drug waste, poor solubility, and damage to other organs. Therefore, in addition to the efficacy of the prepared NPs, we must consider the biodegradability, solubility and uptake rate of the drug in the body if we want to translate them clinically. While improving the delivery method to increase the rate of drug uptake, the half-life must be strictly controlled so that the drug can have a strong therapeutic effect as well as its own degradation to prevent irreversible damage to the liver and kidneys in the long-term [161].

Nanomedicines have shown great potential in ALI therapy due to their advantages of slow release, controlled release, good targeting, good distribution, and few side effects. Nanomaterials need to be continually updated as research into mechanisms deepens. Exploring this research will require more collaboration and effort from researchers from different fields and backgrounds. Although there may be some key challenges and issues, advances in science and technology may facilitate the clinical translation of targeted therapies for ALI.

## Data Availability

No datasets were generated or analysed during the current study.

## Abbreviations

ALI: Acute lung injury
EnaC: Epithelial sodium channel
PMN: Polymononuclear cell
RBC: Red blood cell
TBSA: Total body surface area
TNF-α: Tumor necrosis factor-alpha
IL-1: Interleukin-1
IL-6: Interleukin-6
IL-4: Interleukin-4
IL-10: Interleukin-10
IL-13: Interleukin-13
IL-8: Interleukin-8
ASASs: Alveolar surface-active substances
NPs: Nanoparticles
ELVIS: Extravasation through leaky vasculature and subsequent inflammatory cell-mediated sequestration
ECs: Endothelial cells
PPy: Polypyrrole
DIP: Dipyrrole
TLR4: Toll-like receptor 4
NETs: Neutrophilulartraps
MMP-9: Matrix metalloproteinase 9
LPS: Lipopolysaccharide
Nrf2: Nuclear factor erythroid2-related factor 2
HO-1: Heme oxygenase 1
NQO1: NAD(P)H dehydrogenase quinone 1
ROS: Reactive oxygen species
Bcl-2: B-cell lymphoma-2
AMs: Macrophages
mtDNA: Mitochondrial DNA
DAMPs: Damage-associated molecular patterns
MTDs: Mitochondrial damage-associated molecular patterns
OXPHOS: Oxidative phosphorylation
DCs: Dendritic cells
